# It’s who, not what that matters: personal relevance and early face processing

**DOI:** 10.1093/scan/nsad021

**Published:** 2023-04-20

**Authors:** Mareike Bayer, Tom Johnstone, Isabel Dziobek

**Affiliations:** Berlin School of Mind and Brain, Humboldt-Universität zu Berlin, Berlin 10099, Germany; Clinical Psychology of Social Interaction, Department of Psychology, Institute of Life Sciences, Humboldt-Universität zu Berlin, Berlin 10099, Germany; Centre for Integrative Neuroscience and Neurodynamics, School of Psychology and Clinical Language Sciences, The University of Reading, Reading, UK; School of Health Sciences, Swinburne University of Technology, Hawthorn, Victoria 3184, Australia; Berlin School of Mind and Brain, Humboldt-Universität zu Berlin, Berlin 10099, Germany; Clinical Psychology of Social Interaction, Department of Psychology, Institute of Life Sciences, Humboldt-Universität zu Berlin, Berlin 10099, Germany

**Keywords:** personal relevance, faces, emotion, familiarity, ERPs

## Abstract

The faces of our friends and loved ones are among the most pervasive and important social stimuli we encounter in our everyday lives. We employed electroencephalography to investigate the time line of personally relevant face processing and potential interactions with emotional facial expressions by presenting female participants with photographs of their romantic partner, a close friend and a stranger, displaying fearful, happy and neutral facial expressions. Our results revealed elevated activity to the partner’s face from 100 ms after stimulus onset as evident in increased amplitudes of P1, early posterior negativity, P3 and late positive component, while there were no effects of emotional expressions and no interactions. Our findings indicate the prominent role of personal relevance in face processing; the time course of effects further suggests that it might not rely solely on the core face processing network but might start even before the stage of structural face encoding. Our results suggest a new direction of research in which face processing models should be expanded to adequately capture the dynamics of the processing of real-life, personally relevant faces.

## Introduction

Face familiarity has a large impact on face processing: familiar faces can be detected and recognized in a highly robust, quick and effortless way (Ramon and Gobbini, 2018). In contrast, it has been shown that recognition of unfamiliar faces is surprisingly error-prone and relies heavily on image matching (Young and Burton, 2017). Next to face familiarity, emotional facial expressions have been shown to have a large impact on face processing. Previous event-related potential (ERP) research has demonstrated increased activity for emotional as compared to neutral facial expressions throughout all stages of processing, starting within 100 ms of stimulus onset (Pourtois *et al.*, 2004; Rellecke *et al.*, 2012; Bayer and Schacht, 2014; Schindler and Bublatzky, 2020). The present study aimed to investigate the time course of personal face relevance and emotional facial expressions, as well as possible interactions between both factors, which have rarely been studied so far.

Even though the most important familiar faces in our lives are those of our relevant others, family and friends, relatively few studies have investigated the impact of genuine personal relevance on face processing. Instead, research on familiar faces has mostly used portraits of famous persons like actors or politicians since the use of one single stimulus set for all participants is convenient and affords a level of experimental control (Ramon and Gobbini, 2018). In contrast, research on personally familiar faces requires the use of individualized stimulus sets, which are less convenient to collect and afford less experimental control. However, research has shown considerable differences between personally relevant and merely familiar faces, suggesting that the effort to investigate personally relevant faces is worthwhile to gain unique insights into real-life face processing (Ramon and Gobbini, 2018). For example, personally relevant faces are ‘learned’ via real-life social interactions and are associated with knowledge about the person’s biography and personality traits, with one’s emotional reactions toward the person and with shared memories (Ramon and Gobbini, 2018). Neuroimaging studies suggest that the superior recognition of personally relevant faces is based on the engagement of an extended face processing network in addition to the well-known core face network. The extended system includes brain regions related to memory retrieval (precuneus and anterior temporal cortex), person knowledge (temporoparietal junction), emotion processing (amygdala and insula) and the monitoring of self-relevant information (medial prefrontal cortex) (Gobbini and Haxby, 2007; Visconti Di Oleggio Castello *et al.*, 2017). In addition, highly relevant faces, like those of loved ones, activate cortical and subcortical structures associated with reward value and motivation (Bayer *et al.*, 2021; Sugiura, 2014).

Electroencephalography (EEG) and ERP studies provide information about the time course of personal relevance processing, which is informative for theoretical models of face processing. Some studies have reported effects of personal relevance to occur from 170 ms after stimulus onset, increasing the amplitude of the N170/M170 component in response to family members (Caharel *et al.*, 2005) and fellow students (Kloth *et al.*, 2006). The N170 component, a temporo-occipital negativity, has been linked to structural face encoding in the fusiform gyrus, but findings are ambiguous concerning whether or not it reflects individual face representations (Horovitz *et al.*, 2004; Eimer, 2011; Rossion and Jacques, 2012). At a later stage, amplitudes of the N250r component, a negativity over inferior temporal electrode sites around 250 ms after stimulus onset, were increased for personally familiar faces (university lecturers) in comparison to famous faces and unfamiliar faces, suggesting increased activity in face recognition units (Herzmann *et al.*, 2004). Finally, ERP studies have frequently reported increased amplitudes in the P3 time range, a centro-parietal positivity starting from 300 ms after stimulus onset, for highly personally relevant faces of partners, parents or friends as compared to strangers and famous faces (Langeslag *et al.*, 2007; Grasso and Simons, 2011; Guerra *et al.*, 2012; Langeslag, 2022). The P3 component not only reflects higher-order processing related to stimulus relevance and evaluation (Verleger, 2020) but has also been related to emotional processing and arousal (Cuthbert *et al.*, 2000). In the latter instances, the component is often called the late positive component (LPC), describing mostly the later part of the long-lasting P3 complex.

So far, no ERP study has reported earlier effects of personal relevance, i.e. before the stage of structural face encoding as reflected by the N170 component. These early, perceptual stages of processing are reflected in the P1 component, an occipital positivity generated in the extrastriate visual cortex around 100 ms after stimulus onset (Hillyard and Anllo-Vento, 1998). Most of the available studies on personally familiar face processing have not analyzed the P1 component (Caharel *et al.*, 2005; Langeslag *et al.*, 2007; Wild-Wall *et al.*, 2008; Grasso and Simons, 2011; Guerra *et al.*, 2011; Langeslag and van Strien, 2019), and others reported no effects (Herzmann *et al.*, 2004; Keyes *et al.*, 2010; Caharel *et al.*, 2014; Langeslag and van Strien, 2020). However, more recently, EEG and magnetoencephalography studies that employed multivariate pattern analysis (MVPA) techniques reported that several aspects of faces, including identity, age and gender might be represented even within 100 ms after stimulus onset (Nemrodov *et al.*, 2016; Vida *et al.*, 2017; Dima *et al.*, 2018). Interestingly, one study showed that encoding of these features might be enhanced for familiar faces (famous actors) (Dobs *et al.*, 2019). These findings are noteworthy since identity-specific information had been assumed to only be available after the stage of structural face encoding as reflected in the N170. The reported effect of face familiarity (Dobs *et al.*, 2019) suggests that the general advantage for familiar faces reported prior might be based on early, perceptual processing even before 170 ms. Therefore, the present study aimed to investigate the time line of personally relevant face processing, including early perceptual processing stages as reflected in the P1.

Besides the personal relevance of faces, emotional facial expressions have been shown to have a large impact on face processing. Previous research has demonstrated increased activity for emotional as compared to neutral facial expressions, starting at the stage of perceptual encoding (Pourtois *et al.*, 2004; Rellecke *et al.*, 2012) throughout higher-order evaluative stages (Bayer and Schacht, 2014; Schindler and Bublatzky, 2020). The occurrence of emotion effects at early, perceptual processing stages is yet another example of rapid information extraction from face stimuli, prior to structural encoding as reflected in the N170. In the case of emotional facial expressions, research has suggested that the amygdala plays a causal role in the amplification and detection of emotional content (Vuilleumier *et al.*, 2004) and is especially sensitive to fearful facial expressions (Vuilleumier and Pourtois, 2007). However, since it was suggested that variant and invariant aspects of faces (like emotions and identity) might rely on distinct neural networks (Bruce and Young, 1986; Kanwisher, 2000), it is conceivable that a region of the brain other than the amygdala might be responsible for the amplification of ‘personal’ relevance. Obvious candidates are regions of the extended face processing network, which include areas associated with rewards and the monitoring of personal relevance (Gobbini and Haxby, 2007).

Importantly, we also investigated possible interactions of personal relevance and emotional facial expressions. Very few studies have previously implemented manipulations of both personal relevance and emotional facial expressions. One study reported increased amplitudes in P3 amplitudes decomposed by principal component analyses in mothers viewing pictures of their own crying infant compared to other infants and expressions (Doi and Shinohara, 2012); another study reported shorter onsets of the lateralized readiness potential for happy *vs* disgusted faces specifically for familiar faces (Wild-Wall *et al.*, 2008). Despite this lack of research, the manipulation of both personal relevance and emotional facial expressions is highly relevant since prevalent models of face perception, such as the models by Bruce and Young (1986) and Haxby *et al.* (2000), assume that identity and emotion, or, in the latter model, stable and variable aspects of faces are (initially) processed via different routes. This is in accordance with a previous report of independent effects of personal relevance and emotional expressions in the N170 (Caharel *et al.*, 2005), whereas there are few reports of interactions at higher-order stages indexed by the P3 (Wild-Wall *et al.*, 2008; Doi and Shinohara, 2012). However, several emotion theories, including appraisal theories (Scherer, 2001) and biological approaches focusing on motivation and emotion (Lang and Bradley, 2010), have posited that enhanced processing of emotional content might be based on its increased relevance for the individual. Since personal familiarity is likely to increase the relevance of an emotional expression, this might also be reflected in increased emotion effects to personally familiar faces even at earlier stages. Such effects have already been shown outside the face domain, e.g. in response to reading statements about relevant others (Bayer *et al.*, 2017). However, the specific direction of an interaction between emotion and identity is likely to depend on the specific context. For example, a personally relevant, positively connotated context could be expected to change the relevance of specific, contextually relevant emotional expressions: in relationships with our loved ones, a smile is likely to carry a richer emotional meaning than the smile of a stranger. On the other hand, the angry or fearful expression of a stranger might elicit higher uncertainty and a stronger alerting reaction than the expression of a loved one.

We presented our participants with portraits of their partner, a male close friend and a stranger, displaying fearful, happy and neutral facial expressions. Both partner and close friend are associated with high personal relevance for the subject, as compared to the stranger. In addition, the partner is usually associated with feelings of romantic love and might thus be of even higher relevance.

We expected increased amplitudes of ERP components for personally relevant faces compared to the stranger’s face. Previous results are equivocal as to the time line of face familiarity: while earliest ERP effects were reported for the N170, MVPA results suggest that face familiarity might impact even earlier stages of face processing.

For emotional facial expressions, we expected to replicate increased amplitudes for happy and fearful facial expressions compared to neutral facial expressions starting at the stage of perceptual processing at around 100 ms. Concerning potential interactions between personal familiarity and emotional expressions as well as their directions, previous evidence is scarce, but we hypothesize that personal relevance will likely increase the relevance of emotional facial expressions, potentially reflected in higher amplitudes of ERP components and higher valence/arousal ratings for emotional facial expressions of relevant others compared to strangers. Concerning the time line of effects, we expected independent effects of personal relevance and emotional expressions on the N170 (Caharel *et al.*, 2005) and interactions in the P3/LPC (Wild-Wall *et al.*, 2008; Doi and Shinohara, 2012).

## Materials and methods

The study was reviewed and approved by the University of Reading Research Ethics Committee and was conducted in accordance with the Declaration of Helsinki. All participants provided written informed consent. The study was performed as a simultaneous EEG-functional magnetic resonance imaging (fMRI) coregistration study. fMRI results and cross-modal representational similarity analyses (RSAs) as well as EEG regression analyses have been previously published (Bayer *et al.*, 2021).

### Participants

We collected 64-channel EEG data from 19 female participants; one dataset had to be discarded due to excessive artifacts. The remaining 18 participants (mean age = 19.8 years, s.d. = 1.0 years) had normal or corrected-to-normal vision. All participants were in a heterosexual relationship at the time of data collection (mean duration = 20.8 months, s.d. = 14 months, range = 6–54 months; mean duration of friendship = 36.9 months, s.d. = 28.7 months, range = 9–96 months). Participants received a mean score of 7.66 points (s.d. = 0.61 points) on the Passionate Love Scale (Hatfield and Sprecher, 1986). In addition, participants indicated how happy/content they were with their relationship and their friendship using a visual analogue scale, reporting high content both with their relationship (mean = 8.8/10, s.d. = 1.2) and with their friendship (mean = 8.2/10, s.d. = 1.3). Participants received £25 for participation; they were recruited via the Undergraduate Research Panel and through internet ads.

Our sample size was limited by cost, feasibility and practical considerations, given the complex experimental EEG-fMRI setup in combination with individualized stimulus sets. We performed sensitivity analyses using MorePower (Campbell and Thompson, 2012). Our final sample size allowed for the detection of main effects (one-factor repeated-measures analysis of variance (ANOVA) with three levels) of η_p_^2^ = 0.236, with at α = 0.05 and a power of 0.80. Similar or higher effect sizes have been reported in the previous literature for the effects of personal relevance (Grasso *et al.*, 2009; Grasso and Simons, 2011; Langeslag and van Strien, 2019) as well as emotion effects (Bayer and Schacht, 2014; Schindler *et al.*, 2021). Concerning interactions of emotional expressions and familiarity (3 x 3 repeated-measures ANOVA), our design provided sensitivity for the detection of an effect size of η_p_^2^ = 0.15, thus being within the range of a previously reported interaction (Doi and Shinohara, 2012).

### Stimuli

Participants were presented with portraits of two personally relevant faces (their partner and a male friend) and a male stranger (the same stranger for all participants), showing fearful, happy and neutral facial expressions in a 3 (Identity) x 3 (Emotion) design. Stimuli were acquired as screen shots during Skype sessions; depicted individuals were directed to look straight at the camera and received guidance by the experimenter in order to display the correct emotional expressions. After acquiring the neutral expression, we used short verbal instructions in order to elicit happy and fearful expressions, e.g. by imagining a frightening or happy situation, and gave feedback on the facial expression when necessary. We took care to provided sufficient time for our participants’ relevant others to feel comfortable with the photo session and to imagine a respective emotional situation. We aimed to collect authentic rather than extreme variants of fearful and happy expressions in order to ensure a naturalistic stimulus set. Pictures were edited to show the complete face (including hair) in color on a gray background. After the experiment, participants rated their individual stimulus set using 7-point Likert scales regarding the valence and arousal of the depicted facial expression, as well as the attractiveness of the depicted person (for the latter, the neutral expression was presented). In order to control for lower-level stimulus features, we conducted control analyses by applying a hierarchical computational model of cortical responses of neurons in the primary visual cortex (HMAX, Serre *et al.*, 2007) to our stimulus pictures. These analyses were performed on the output of the second layer (C1) of the HMAX model and revealed no significant differences in modeled low-level visual stimulus features between experimental conditions, *F*(2,34) < 1, *P* = 0.747, η_p_^2^ = 0.017. For stimulus ratings, see Table 1, and statistics and details on HMAX analyses are reported in the supplementary information.

**Table 1.** Stimulus ratings for attractiveness, valence and arousal

	Attractiveness (1–7), mean (s.d.)	Valence (−3 to +3), mean (s.d.)			Arousal (1–7), mean (s.d.)		
		Fearful	Happy	Neutral	Fearful	Happy	Neutral
Partner	6.2 (1.0)	0.2 (1.6)	2.9 (0.2)	0.7 (1.5)	3.3 (1.8)	5.7 (1.2)	4.1 (1.6)
Friend	4.2 (1.4)	−0.7 (1.2)	2.1 (0.8)	−0.1 (1.2)	2.1 (1.5)	3.1 (2.0)	2.3 (1.6)
Stranger	4.2 (1.2)	−1.4 (1.1)	1.1 (1.1)	0.0 (1.2)	1.7 (1.2)	3.2 (2.0)	2.6 (1.7)

### Procedure

After signing informed consent, participants were fitted with the EEG cap and placed in the MRI scanner. Participants performed a passive face-viewing task with occasional one-back trials in order to keep participants engaged in the task. Face stimuli were presented for 1 s, followed by a variable intertrial interval (ITI) with a mean ITI of 3 s and a minimum ITI of 2.5 s, sampled using an exponential function using the program fMRI Simulator (Rorden, 2011). During the ITI, a central fixation cross was presented. Every face was presented 40 times in a pseudo-randomized order (also determined by the fMRI Simulator), resulting in 360 experimental trials. In addition, 40 one-back trials were presented. These trials were identified by a centrally presented question mark, which was followed by a face stimulus. Participants had to decide by pressing a button whether the face following the question mark was identical (in emotion and identity) to the one presented directly before the question mark. Stimuli were presented in 10 blocks of 40 stimuli each, with short breaks in between blocks. Stimuli were presented on a Nordic Neuro Labs goggle system at 60 Hz on an 800 × 600 pixel screen using E-Prime software (Psychology Software Tools, Inc.).

### Data acquisition and preprocessing

EEG data were acquired using an MRI-compatible system (Brain Products) with 64 Ag–AgCl electrodes, and the electrocardiogram (ECG) was recorded from an electrode placed left of the spinal column. Electrode impedances were kept <20 kΩ. Data were sampled with 5000 Hz with electrodes FCz as reference and AFz as ground and high-pass filtered at 0.1 Hz. EEG and fMRI clocks were synchronized using a sync box (Brain Products) in order to align EEG recordings and MRI slice acquisition.

Offline, MR gradient artifacts were identified using synchronized scanner markers and removed from continuous EEG data with a template subtraction algorithm using a sliding window of 21 artifacts on baseline-corrected data (whole artifacts used for baseline correction) (Allen *et al.*, 2000). Data were re-referenced to average reference, downsampled to 250 Hz and low-pass filtered using a finite impulse response filter (70 Hz). Ballistocardiographic artifacts were identified using the ECG channel with a semiautomatic template matching procedure and corrected using a template subtraction approach (sliding window of 15 pulse intervals). Eye blinks, eye movements and residual ballistocardiographic artifacts were removed using a restricted InfoMax independent component analysis (Bell and Sejnowski, 1995). Data were segmented into epochs from −100 ms before stimulus onset to 800 ms after stimulus onset and baseline-corrected using a 100 ms pre-stimulus baseline. Trials with activity exceeding ±100 μV or voltage steps >100 μV were excluded from analyses (0.6 % of trials). There were no significant differences between trial numbers per condition as assessed with a repeated-measures ANOVA (3 Identity x 3 Emotions); all *F*s < 2.11, all *P*s > 0.136; descriptive statistics on trial numbers per condition are listed in Supplementary Table S2. Data were averaged per participant and experimental condition.

### Data analyses

Analyses were performed on ERP components P1, N170, P3 and LPC. P1 amplitudes were quantified at the average of occipital electrodes PO8, PO4, POz, PO3, PO7, O1, Oz and O2 using a semi-automatic peak detection algorithm in the time window from 90 to 130 ms after stimulus onset (mean peak latency = 107 ms). N170 amplitudes were detected at averaged electrodes TP9, TP7, TP8, TP10, P7 and P8 in the time window from 150 to 220 ms after stimulus onset (mean peak latency = 172 ms).

P3 and LPC amplitudes were analyzed as the average of centro-parietal electrodes CP1, CPz, CP2, P1, Pz, P2 and POz in the time window of 300 to 400 ms (P3) and 400–800 ms (LPC). In addition, we performed exploratory analyses of the early posterior negativity (EPN), a negativity at temporo-occipital electrode sites frequently reported for emotional *vs* neutral facial expressions (Bayer and Schacht, 2014), which was quantified at the average of temporo-parietal electrodes TP9, TP10, P7, P8, PO7 and PO8 in the time window of 200–300 ms.

Analyses were performed in JASP (JASP Team, 2021) with repeated-measures ANOVAs including the factors Identity (3) and Emotion (3). Huynh–Feldt correction was applied for violations of sphericity; *post hoc* tests were Bonferroni-corrected for multiple comparisons.

In order to follow up on hypothesized but non-significant main effects, we performed exploratory Bayesian repeated-measures ANOVAs (using a multivariate Cauchy prior) including the factors Identity (3) and Emotion (3) in order to quantify the level of support for the null hypothesis, i.e. for a null model that assumes no differences between conditions, compared to a model that includes such differences (note that all models include the factor subject). We report BF_01_, i.e. the Bayes factor that quantifies evidence for the null hypothesis, with values >1 indicating evidence for H0 over H1 (JASP Team, 2021).

Ratings of stimulus valence, arousal and attractiveness were analyzed with repeated-measures ANOVAs in parallel to ERP analyses (3 x 3 for valence and arousal ratings; analyses of attractiveness ratings only contained the factor Identity). Planned *post hoc* tests for ratings of valence and arousal were conducted as analyses of Identity effects within each emotion category, followed up by pairwise comparisons in case of significant effects.

## Results

### ERPs

#### P1

**Fig. 1.**
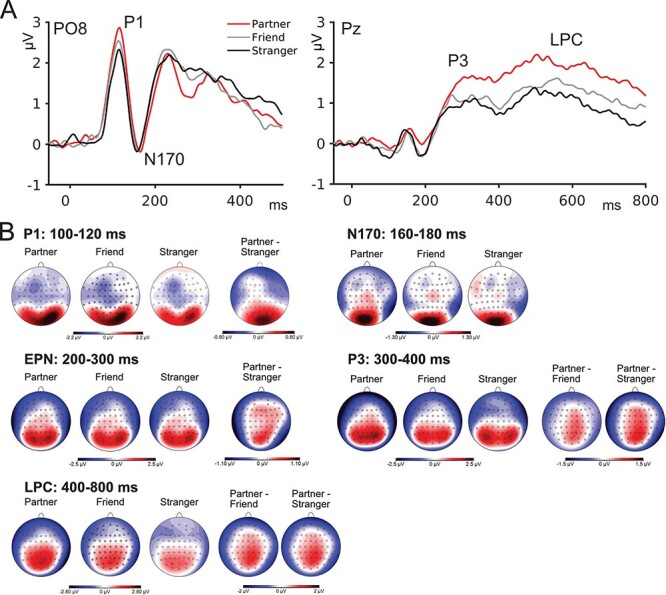
Effects of personal relevance in ERPs. Grand mean ERP waveforms for Partner, Friend and Stranger at electrodes PO8 and Pz (A). Scalp distributions for Partner, Friend and Stranger, as well as difference topographies for indicated ERP components and time intervals (B).

Analyses of P1 peak amplitudes revealed a main effect of Identity, *F*(2,34) = 3.80, *P* = 0.031, and η_p_^2^ = 0.183, based on larger amplitudes for Partner compared to Stranger, *t* = 2.71, *P *= 0.031, and *Cohen’s d* = 0.640; there were no significant effects for the comparisons of Partner *vs* Friend and Friend *vs* Stranger, all *P*s > 0.22. Furthermore, there was no significant effect of Emotion, *F*(2,34) < 1, and no significant interaction of Emotion and Identity, *F*(4, 68) = 1.21 and *P* = 0.315. See Figure 1 for waveforms and topographies.

#### N170

For N170 amplitudes, no effects of Identity, Emotion or any interactions were observed, all *F*s < 1.53 and all *P*s > 0.21.

#### P3 (300–400 ms)

Analyses of P3 amplitudes showed a significant effect of Identity, *F*(2,34) = 12.61, *P* < 0.001, and η_p_^2^ = 0.426. The effect was based on larger amplitudes for Partner compared to Friend, *t* = 3.452, *P* = 0.005, and *Cohen’s d* = 0.814, and to Stranger, *t* = 4.885, *P* < 0.001, and *Cohen’s d* = 1.151; there was no significant difference between Friend and Stranger. No significant effects emerged for Emotion or the interaction of Emotion and Identity, all *F*s < 1.48 and all *P*s > 0.244.

#### LPC (400–800 ms)

As for the P3, analyses of the LPC revealed an effect of Identity, *F*(2,34) = 18.76, *P* < 0.001, and η_p_^2^ = 0.525. Again, amplitudes were increased for Partner compared to Friend, *t* = 3.32, *P* = 0.007, and *Cohen’s d* = 0.781, and to Stranger, *t* = 6.12, *P* < 0.001, and *Cohen’s d* = 1.442. Finally, LPC amplitudes were also increased for Friend compared to Stranger, *t* = 2.803, *P* = 0.025, and *Cohen’s d* = 0.661. No significant effects were observed for Emotion or the interaction of Emotion and Identity, *F*s < 2.02 and *P*s > 0.148.

### Exploratory analyses

#### EPN

Analyses of EPN amplitudes revealed a main effect of Identity, *F*(2,34) = 5.38, *P* = 0.021, and η_p_^2^ = 0.24, with increased, i.e. more negative-going, amplitudes for Partner than Stranger, *t* = −3.21, *P* = 0.009, and *Cohen’s d* = −0.758, whereas amplitudes did not differ for Partner *vs* Friend and Friend *vs* Stranger. The main effect of Emotion was not significant, *F*(2,34) = 2.98 and *P* = 0.057, and there was no significant interaction between Identity and Emotion, *F*(4,68) = 1.52 and *P* = 0.222.

#### Bayesian analyses of main effects

For P1 amplitudes, Bayesian repeated-measures ANOVA revealed a BF_01_ = 6.67 for the factor emotion, indicating that the data are 6.67 times more likely under a null model than under a model that assumes differences between emotion categories. For the N170, analyses revealed a BF_01_ = 15.67 for the factor Emotion and 1.81 for Identity. Likewise, Bayes factors for the other components showed evidence for the null model over models including the factor Emotion, with BF_01_ = 10.17 (EPN), BF_01_ = 5.31 (P3) and BF_01_ = 4.79 (LPC). In summary, while our data revealed significant main effects of Identity, Bayesian analyses showed strong and consistent evidence for models that assume no effect of the factor Emotion.

### Stimulus ratings

**Fig. 2.**
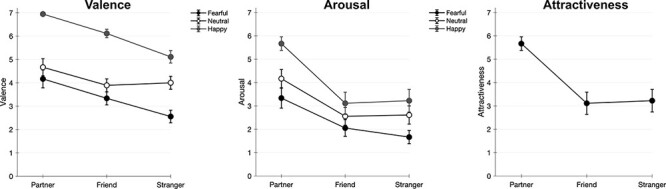
Stimulus ratings of valence, arousal and attractiveness (means and standard errors). Attractiveness ratings were collected for neutral facial expressions.

For a complete list of statistical details, please see Supplementary Table S1. Here, we focus on main effects and the description of (all) significant interactions. For attractiveness ratings, there was a main effect of Identity, *F*(2,34) = 30.58, *P* < 0.001, and η_p_^2^ = 0.643, based on higher ratings for Partner compared to Friend and Stranger (see Figure 2). For valence ratings, we observed main effects of Identity, *F*(2,34) = 14.45, *P* < 0.001, and η_p_^2^ = 0.462, with more positive ratings for Partner compared to Friend and Stranger, as well as a main effect of Emotion, *F*(2,34) = 59.26, *P* < 0.001, and η_p_^2^ = 0.777: as expected, valence ratings were highest for happy facial expressions, intermediate for neutral expressions and lowest for fearful expressions. Finally, there was an interaction of Identity and Emotion, *F*(4,68) = 5.43, *P* < 0.001, and η_p_^2^ = 0.242. Within fearful faces, the Partner was rated as more positive than Stranger; within happy faces, the Partner received more positive ratings than Friend and Stranger, and Friend was rated more positive than Stranger. Within neutral faces, there was no effect of Identity.

For arousal ratings, we also found a main effect of Identity, *F*(2,34) = 24.39, *P* < 0.001, and η_p_^2^ = 0.589, based on higher arousal ratings for Partner compared to Friend and Stranger. A main effect of Emotion, *F*(2,34) = 13.25, *P* < 0.001, and η_p_^2^ = 0.438, was based on higher arousal ratings for happy faces compared to neutral and fearful faces. Finally, there was an interaction of Identity and Emotion *F*(4,68) = 3.33, *P *< 0.029, and η_p_^2^ = 0.164; however, planned *post hoc* tests revealed higher arousal ratings for Partner compared to Friend and Stranger in all emotional facial expressions.

## Discussion

We investigated the impact of personal relevance and emotional facial expressions on the time course of face processing using ERPs. Seeing the face of one’s partner compared to a stranger increased the amplitudes of the P1 component at 100 ms after stimulus onset, even before the stage of structural face encoding. While no effects of Identity were observed during structural encoding as indicated by the N170, later, evaluative stages showed pronounced effects of Identity, reflecting the degree of personal relevance. In contrast, we observed no effects of emotional facial expressions, suggesting the paramount impact of personal relevance in everyday face processing.

The finding of increased P1 amplitudes in response to the Partner’s face is especially noteworthy since it is related to perceptual processing at around 100 ms after stimulus onset in the extrastriate cortex and thus precedes structural face encoding as indexed by the N170 component (Eimer, 2011), as well as subsequent face identification processes (Bruce and Young, 1986). However, our results are not necessarily in conflict with established face processing models. Instead, we suggest the involvement of additional mechanisms in the processing of personally familiar faces. Indeed, neuroimaging studies have identified an extended face network that includes regions related to reward processing, memory, self-relevance monitoring and emotions (Gobbini and Haxby, 2007; Visconti Di Oleggio Castello *et al.*, 2017; Ramon and Gobbini, 2018). The time course of our effects suggests that at least some of these regions might enable the early amplification of personal relevance already at the stage of perceptual encoding in the extrastriate visual cortex. Similar early effects have been shown for faces and other stimuli with emotional and motivational relevance (Pourtois *et al.*, 2013). Furthermore, these effects have been related to associative learning, linking the physical stimulus features with the valence or reward value of a given stimulus (Steinberg *et al.*, 2013; Rossi *et al.*, 2017; Bayer *et al.*, 2019), with involvement of prefrontal regions (Rehbein *et al.*, 2014). These assumptions are corroborated by previous results on the role of prefrontal areas in personally familiar face processing (Karimi-Rouzbahani *et al.*, 2021) and also by the results of the EEG-informed fMRI analyses using RSA we performed on the current data (Bayer *et al.*, 2021), which are consistent with early representations around 100 ms after stimulus onset not only in sensory areas but also in the prefrontal cortex (Bayer *et al.*, 2021). On a theoretical level, these data and the current ERP results are consistent with the interactive activation model of face processing (Burton *et al.*, 1990), which suggests that additional semantic information about a person (which is available for personally relevant others) facilitates face perception. This is also in line with faster and more robust recognition of familiar faces reported in the previous literature (Young and Burton, 2017; Ramon and Gobbini, 2018). However, based on our data we cannot distinguish whether additional information and/or neural mechanisms are qualitatively or quantitatively different for different types of face familiarity (for discussion, see Wiese *et al.*, 2021).

The stage of structural encoding, as captured in the N170 component, did not show modulations by Identity or Emotion, or their interaction. For both Identity and Emotion effects, previous research is characterized by pronounced heterogeneity concerning modulations of the N170. In the case of emotional expressions, it has previously been suggested that the N170 might be affected by overlapping but independent of emotion-related processing (Rellecke *et al.*, 2013). Concerning effects of Identity, the heterogeneity remains a matter of debate, with some studies reporting effects (Caharel *et al.*, 2005, 2014), while others do not (Eimer, 2011; Doi and Shinohara, 2012; Guerra *et al.*, 2012; Langeslag and van Strien, 2020; Schindler *et al.*, 2021). Our data, together with previous studies, provide a tentative indication that the core face processing network might not necessarily be involved in the processing of personal relevance during perceptual and structural encoding stages, in line with established models of face processing (Bruce and Young, 1986).

At the stage of higher-order processing after 300 ms from stimulus onset, reflected in the amplitudes of the P3 and LPC, personally relevant faces of the Partner elicited considerably increased amplitudes compared to both Friend and Stranger, as well as for Friend compared to Stranger. These findings are in line with previous reports (Langeslag *et al.*, 2007; Guerra *et al.*, 2012) and show that P3 amplitudes reflect the ‘degree’ of personal relevance (Vico *et al.*, 2010), thus demonstrating the importance of including highly personally relevant faces, instead of or in addition to less relevant or famous faces. This is also suggested by neuroimaging studies showing additional activations in response to personally relevant faces in brain structures related to reward, emotion and motivational value (Sugiura, 2014).

Finally, it is noteworthy that in the present study, we report no ERP effects of emotional facial expressions, while personal relevance was strongly reflected in our ERP data (however, note that the effects of emotional facial expressions were evident in unimodal fMRI analyses, see Bayer *et al.* (2021), corroborating previous findings of emotion effects and indicating that the absence of emotion effects in the present ERP data might not solely be attributed to the stimulus material and its potential to elicit emotion effects). Even the EPN, a component typically associated with facilitated sensory processing of emotional compared to neutral stimulus content, (see also Langeslag, 2022), was increased by personal relevance but did not show effects of emotions. The absence of emotion effects in our ERP analyses was further corroborated by Bayesian analyses showing evidence for a null model without differences between emotion categories for all investigated ERP components, thus suggesting that our null findings were not based on insufficient statistical power to detect such effects. These findings suggest that emotional facial expressions, and social stimuli like faces more generally, are not processed in an automatic fashion. Instead, brain responses seem to reflect context-specific importance of social stimuli: in our experimental design, seeing the partner’s face is likely more relevant than any additional information conveyed by the (context-free) emotional facial expression. Furthermore, this context-specificity might explain a more general variability and heterogeneity in research findings on familiar face processing: in our study, we observed strong and early effects for Partner, whereas differential effects for Friend *vs* Stranger were only visible during the LPC time window. This is in contrast to some previous studies that reported earlier effects in response to friends’ faces (Herzmann *et al.*, 2004; Caharel *et al.*, 2006; Wiese *et al.*, 2021). However, it is noteworthy that these studies did not include another potentially more relevant or salient face condition like the participant’s partner. Furthermore, and in line with our findings, previous studies that included the same experimental conditions (Partner, Friend, and Stranger) also reported very few and inconsistent effects for Friend *vs* Stranger in the observed time intervals (Langeslag *et al.*, 2007; Langeslag and van Strien, 2019, 2020). A similar issue emerges when considering why other studies investigating the P1 component in response to personally relevant faces did not report significant effects of personal relevance (Herzmann *et al.*, 2004; Keyes *et al.*, 2010; Caharel *et al.*, 2014; Langeslag and van Strien, 2020). A possible explanation arises when considering the experimental designs of these other studies: for example, three of these studies did not include a partner condition but rather presented university lecturers (Herzmann *et al.*, 2004), fellow students (Caharel *et al.*, 2014) or friends (Keyes *et al.*, 2010) as relevant others. In another study, participants were presented with pictures of their partner but merely as task-irrelevant stimuli embedded in a memory task (Langeslag and van Strien, 2020). Taken together, these findings seem to suggest that the relevance of facial stimuli, concerning both their emotional expression and their (degree of) familiarity, seems to be decoded in a context-specific manner. Furthermore, our results present a compelling case for including stimuli tailored to the individual in future research in order to investigate real-life face processing, instead of relying on standardized datasets of faces with little personal relevance.

Finally, it is noteworthy that even though ERP data did not reveal effects of emotion, stimulus ratings of valence and arousal did show effects of both emotion and personal relevance, as well as interactions of the two factors. As expected, valence ratings showed higher ratings for happy *vs* neutral *vs* fearful faces. However, there was also a main effect of Identity, based on higher valence ratings for Partner compared to both Friend and Stranger. Similarly, arousal ratings were increased for Partner compared to both Friend and Stranger. These findings are in line with previously reported findings of increased valence and arousal for relevant others (Doi and Shinohara, 2012; Langeslag and van Strien, 2019), which even seem to generalize to relevant context information (Langeslag *et al.*, 2015; Bayer *et al.*, 2017). Unfortunately, we did not have ethics committee approval to collect rating data from an independent group of participants. These ratings would have been helpful in distinguishing true effects of personal relevance from potential stimulus characteristics, e.g. concerning the strength of emotional expressions. For example, in our data, fearful faces were rated as more negative than neutral expressions, but not as more arousing. Instead, happy expressions were rated as more arousing (and pleasant) than fearful and neutral faces. This might be due to the more ‘positive’ interpretation of arousal in the context of evaluating pictures of one’s partner; however, we cannot exclude differences in stimulus materials.

This point is also relevant for interactions between Identity and Emotion. In our data, valence ratings were increased for Partner compared to other identities only in fearful and happy expressions, whereas there was no effect of Identity within neutral expressions. These findings are in line with our hypothesis, showing increased effects of emotional facial expressions for relevant others compared to a stranger’s face. Even though interactions between Identity and Emotion have been previously reported for rating data (Doi and Shinohara, 2012), it would be highly interesting for future research to investigate such effects by comparing ratings of relevant others and an independent sample. Finally, participants also rated their partners as more attractive than their friends and the stranger. Again, we cannot exclude stimulus-based differences in ‘objective’ attractiveness (which would have been corroborated by an independent sample). However, our result is in line with a previous study that also reported higher attractiveness ratings for the partner compared to a stranger (and friend), even though the stranger had been composed by blending faces previously rated as beautiful. Furthermore, an independent sample showed the opposite effect and rated the stranger as significantly more attractive than the partners and friends (Langeslag *et al.*, 2007). This shows that, perhaps unsurprisingly, people tend to perceive their own partner as more attractive than they perceive others, independent of the partner’s ‘objective’ average attractiveness.

Taken together, our results demonstrate the strong impact of personal relevance on face processing, most likely based on the involvement of brain regions outside of the core face processing network. These effects occur as early as 100 ms after stimulus onset in the extrastriate visual cortex, as well as in higher processing stages. Our study also shows the importance to include individualized stimulus sets in order to capture our real-life social interactions.

## Supplementary Material

nsad021_Supp

## Data Availability

Data are available at https://osf.io/gzsfu/?view_only=d6e5960795b04d218f32117267de9e1e.
