# Supplementary material for: It’s who, not what that matters: personal relevance and early face processing
Source: Soc Cogn Affect Neurosci. 2023 Apr 20;18(1):nsad021. doi: 10.1093/scan/nsad021 (PMC10176112; doi:10.1093/scan/nsad021)
Supplement: nsad021_Supp [file nsad021_supp.zip › scan-22-101-File004.docx]

**Supplementary information**

**HMAX-Analyses on visual stimulus features**

In order to control for low-level visual features of our stimulus set, we conducted representational similarity analyses (RSA^1^) based on a computational model of cortical responses in the primary visual cortex (HMAX^2^), more specifically on the output of the second layer C1. Representational dissimilarity matrices (RDMs) were constructed for each participant’s individual stimulus set using Pearson’s correlations. In order to investigate possible differences between experimental conditions, we extracted mean dissimilarities for partner vs. friend, friend vs. stranger and partner vs. stranger for each subject, and performed a repeated-measures ANOVA on the dissimilarity values. Results revealed no significant differences between identities (partner, friend, stranger), *F*(2,34) < 1, *p* = .747, *η_p_^2^*= 0.017.

**Table S1**: **Results of stimulus ratings.** Results of repeated-measures ANOVAs with factors Emotion (Fear, Happy, Neutral) and Identity (Partner, Friend, Stranger). Post-tests were corrected for multiple comparisons using Bonferroni-corrections. Degrees of freedom: F(2,34) for main effects of Attractiveness, Identity and Emotion; F(4,68) for Interactions.

| **Rating** |  | **Identity** | **Emotion** | **Identity x Emotion** |
| --- | --- | --- | --- | --- |
| Attractiveness | *F p η_p_^2^* | 30.58  < .001  .643  Partner > Friend  *t* = 6.86 *p* < .001  Partner > Stranger  *t =* 6.68, *p* < .001  Friend vs. Stranger n.s. | n.a. | n.a. |
| Valence | *F p η_p_^2^* | 14.56  < .001  .462  Partner > Friend  *t* = 3.19*, p = .*009  Partner > Stranger  *t =* 5.37, *p <* .001  Friend vs. Stranger n.s. | 59.26  < .001  .777  Happy > Neutral  *t =* 7.35, *p <* .001  Happy > Fear  *t* = 10.63, *p* < .001  Neutral > Fear  *t =* -3.28*, p = .*007 | 5.43  < .001  .242  Fearful faces: effects of Identity  *F*(2,24) = 14.21, *p* < .001, *η_p_^2^ = .456*  Partner vs. Friend n.s.  Partner > Stranger  *t* = 5.33, *p* < .001  Friend vs. Stranger n.s.  Happy faces: effects of Identity:  *F*(2,34) = 32.92, *p* < .001, *η_p_^2^ = .659*  Partner > Friend *t* = 3.68, *p* = .006  Partner > Stranger *t* = 8.10, *p* < .001  Friend > Stranger  *t* = 4.42, *p* < .001  Neutral faces: effects of Identity n.s. |
| Arousal | *F p η_p_^2^* | 24.39  < .001  .589  Partner > Friend  *t =* 5.92*, p <* .001  Partner > Stranger  *t =* 6.17*, p < .*001  Friend vs. Stranger n.s. | 13.25  < .001  .438  Happy > Neutral  *t = 2.77, p = .*027  Happy > Fear  *t =* 2.37*, p < .*001  Fear vs. Neutral n.s. | 3.33  .029  .164 Fearful faces: effects of Identity  *F*(2,34) = 17.05, *p* < .001, *η_p_^2^* = .501  Partner > Friend  *t* = 4.28, *p* < .001  Partner > Stranger *t* = 5.58, *p* < .001  Friend vs. Stranger n.s.  Happy faces: effects of Identity  *F*(2,34) = 26.48. *p* < .001, *η_p_^2^ = .609*  Partner > Friend  *t* = 6.44, *p* < .001  Partner > Stranger  *t* = 6.12, *p* < .001  Friend vs. Stranger n.s.  Neutral faces: effects of Identity:  *F*(2,34) = 9.05, *p*  < .001, *η_p_^2^ = .347*  Partner > Friend  *t* = 3.75, *p* = .002  Partner > Stranger  *t* = 3.62, *p* = 003  Friend vs. Stranger n.s. |

**Table S2: Descriptive statistics for EEG trial numbers per condition included in ERP analyses.**

| **Condition** | **Mean** | **Min** | **Max** | **Standard Deviation** |
| --- | --- | --- | --- | --- |
| Partner fear | 38.6 | 27 | 40 | 3.2 |
| Partner happy | 38.7 | 28 | 40 | 3.0 |
| Partner neutral | 38.8 | 31 | 40 | 2.7 |
| Friend fear | 39.1 | 30 | 40 | 2.6 |
| Friend happy | 38.8 | 30 | 40 | 3.0 |
| Friend neutral | 38.7 | 24 | 40 | 3.9 |
| Stranger fear | 38.9 | 30 | 40 | 2.5 |
| Stranger happy | 39.2 | 30 | 40 | 2.6 |
| Stranger neutral | 39.0 | 27 | 40 | 3.1 |

1. Kriegeskorte, N. Representational similarity analysis – connecting the branches of systems neuroscience. *Frontiers in Systems Neuroscience* (2008) doi:10.3389/neuro.06.004.2008.

2. Serre, T., Oliva, A. & Poggio, T. A feedforward architecture accounts for rapid categorization. *PNAS* **104**, 6424–6429 (2007).
